# Low incidence of cytolysin-positive *E. faecalis* and no correlation to survival in Danish patients with alcohol-associated hepatitis: A prospective cohort study

**DOI:** 10.1080/29933935.2025.2549729

**Published:** 2025-09-03

**Authors:** Frederik Cold, Julie Elm Heintz, Khaled Saoud Ali Ghathian, Poul Als Stenbøg, Lars Hestbjerg Hansen, Alexander Byth Carstens, Andreas Munk Petersen, Sofie Ingdam Halkjaer, Flemming Bendtsen, Henriette Ytting

**Affiliations:** a Gastro Unit, Medical Section, Copenhagen University Hospital Hvidovre, Hvidovre, Denmark; b Department of Gastroenterology and Hepatology, Copenhagen University Hospital - Gentofte and Herlev, Herlev, Denmark; c Department of Clinical Microbiology, Copenhagen University Hospital Hvidovre, Hvidovre, Denmark; d Department of Plant and Environmental Sciences, University of Copenhagen, Frederiksberg, Denmark; e Department of Clinical Medicine, Faculty of Health and Medical Sciences, Copenhagen University, Copenhagen, Denmark

**Keywords:** Alcoholic hepatitis, alcohol-associated hepatitis, mortality, microbiome, microbiota, cytolysin, *Enterococcus*, *Enterococci*

## Abstract

Alcohol-associated hepatitis (AH) is a severe and life-threatening form of alcohol-associated liver disease with no approved treatments for reducing long-term mortality. Cytolysin-producing *E. faecalis* in the gut microbiota of AH patients has been reported as highly correlated to mortality. We investigated whether we could reproduce this correlation in a cohort of Danish patients with AH. Fecal samples from 28 hospitalized patients with AH were analyzed for cytolysin-producing *E. faecalis* and were followed for 1 y after hospital admission. The primary endpoint was comparison of 180-d mortality in AH patients with and without cytolysin-positive fecal samples. Three of twenty-eight (10.7%) fecal samples were identified as cytolysin-positive. There were no significant differences at baseline between cytolysin-positive and -negative patients in terms of age, Glasgow Alcoholic Hepatitis Score, Charlson Comorbidity Index or biochemical variables (INR, bilirubin, albumin). There was no difference in mortality between the groups 180 d after hospital admission; one of the three (33%) cytolysin-positive patients had died compared to 9 of the 25 (36%) cytolysin-negative (*p*-value for difference = 1.0). We report a low incidence of cytolysin-positive *E. faecalis* in hospitalized Danish AH patients and no greater risk of mortality compared to cytolysin-negative AH patients.

## Introduction

Alcohol-associated hepatitis (AH) is a severe and life-threatening form of alcohol-associated liver disease (ALD) caused by long- or short-term excessive alcohol consumption.^1^ AH is characterized by a sudden onset of jaundice and clinical signs of hepatic decompensation, malaise, right upper abdominal pain representing tender hepatomegaly, fever, and laboratory signs of mild to moderate hepatocyte injury and systemic inflammatory response.^2^ The precise mechanisms behind AH are not fully understood and probably include environmental, genetic, and epigenetic factors.^3,4^

The rate of hospitalization due to AH has increased in recent decades.^1,2^ The mortality of hospitalized patients with severe AH is 20–50% at 90 d.^5,6^ Current treatments include corticosteroids and have been reported to reduce only short-term mortality.^7–9^ Thus, new treatments are needed.

It has been reported that changes in the gut microbiome are related to the development and severity of AH.^10,11^ Excessive alcohol intake induces profound changes in the gut microbiome and disrupts the tight junctions between the intestinal epithelial cells.^12^ Furthermore, excessive alcohol intake leads to the translocation of bacterial DNA into the hepatic circulation in individuals with alcohol-related liver disease.^13^ Translocation of bacteria and microbial products to the liver induces inflammation that, in genetically predisposed individuals, may lead to AH.^1,14^

Modulating the gut microbiome has thus been proposed as a potential treatment approach^15^ and the transfer of intestinal microbiota from healthy donors through fecal microbiota transplantation (FMT) has shown promising results in ALD^16^ and a reduction of 90-d mortality without side effects.^6^

In a breakthrough publication in 2019, Duan et al.^17^ reported that the presence of two cytolysin genes encoded by *Enterococcus (E) faecalis* in patient stool samples were highly correlated to mortality in patients with AH, where 89% of cytolysin-positive patients died within 180 d compared to just 3.8% of cytolysin-negative patients. Moreover, precise targeting of cytolysin-positive (cytolytic) *E. faecalis* with bacteriophage treatment was found to alleviate ALD in a mouse model, and could be a promising therapeutic target for patients with ALD. The correlation between cytolysin-positive *E. faecalis* and mortality in AH has not yet been assessed in a new cohort of patients with AH.

We sought to reproduce the findings of a strong correlation between cytolysin-positive *E. faecalis* in fecal samples and increased mortality in a cohort of hospitalized Danish patients with AH.

## Materials and methods

### Study design

The BATTLE (BActeriophages To Treat Liver disease Eliminating harmful bacteria) study is a prospective cohort study of 30 hospitalized patients with AH. There was no intervention in the study. Participating patients consented to the collection of fecal samples and medical and biochemical data. Patients were enrolled consecutively from June 2022 to September 2023. Enrolment was performed at the Departments of Gastroenterology at the Copenhagen University Hospitals in Hvidovre and Herlev. Both are tertiary care hospitals in the Capital Region of Denmark covering a total population of approximately 1,050,000 individuals.

### Patients

Hospitalized adult patients (age 18 or older) with AH were invited to participate in the BATTLE study. Inclusion criteria for AH were a history of patient-reported excessive alcohol ingestion (> 40/60 g in females/males per day) within the past 3 months and acute jaundice within the past 4 weeks with serum bilirubin >50 mmol/L).^1^ Exclusion criteria were hepatocellular carcinoma, obstruction of bile ducts, viral hepatitis, autoimmune liver disease, complete portal thrombosis, pregnancy, or end-stage liver disease or other diseases with an expected survival of less than 12 months, assessed by the clinician at the trial sites. Obstructive causes of jaundice were excluded through ultrasound (US), computed tomography (CT), or magnetic resonance imaging of the liver and bile ducts.

### Data collection and study endpoints

For all patients, upon admission we recorded previous and present diseases, age, gender, and current medications. Blood samples were taken as standard work-up for patients admitted with AH: bilirubin, international normalized ratio (INR), albumin, creatinine, platelets, leucocytes, CRP, and alanine-aminotransferase (ALT). All patients underwent an US or CT scan within the first week of their hospitalization. Glasgow Alcoholic Hepatitis Score (GAHS),^18^ Child-Pugh score,^19^ Charlson Comorbidity Index (CCI),^20^ and model for end-stage liver disease (MELD) score^21^ were calculated based on values from hospital admission. Cirrhosis was registered if it appeared in a patient’s health record prior to admission or if found during US or CT scan by a radiologist. Diabetes was registered if this was registered in the patient’s health record prior to admission or if the patient received treatment with the following antidiabetic medications: biguanides, sulfonylureas, DPP-4 inhibitors, GLP-1 receptor agonist, thiazolidinediones, or insulin. In the case of treatment with insulin, the type of diabetes registered was based on the physicians’ entry in the patient’s medical record.

### Treatment of complications to liver disease

All patients received standard care according to Danish and international guidelines of treatment of AH.^22^ Severe AH was defined as a GAHS ≥ 9 and treatment with corticosteroids was initiated with oral prednisolone 40 mg daily for 7 d. Following 7 d of treatment, a Lille-score was calculated.^23^ Lille score <0.45 was defined as steroid responsive, and these patients were offered further 21 d of oral prednisolone 40 mg daily. Those with a Lille score ≥0.45 were defined as non-responders to corticosteroids, why prednisolone treatment was stopped.

### Follow-up

From patients’ medical records we collected information about the duration of their primary hospitalization with AH, any intensive care unit (ICU) stays, treatment with antibiotics or corticosteroids, further new hospitalizations and their duration over a 12-month period, and the date and cause of death, if applicable. Specific complications of liver disease – namely, hepatic encephalopathy (HE), ascites, variceal bleeding, and hepatorenal syndrome (HRS) – were registered during the initial hospitalization and at subsequent admissions and follow-up visits. HE and variceal bleeding were registered according to the treating clinician’s notes in the patient’s medical record. Ascites was registered if described by the treating clinician or if found during ultrasound or CT scan. Likewise, HRS was registered if described by the treating clinician, or if a patient’s medical record showed an increase of serum creatinine ≥26.5 µmol/l within 48 h or an >1.5-fold increase from baseline without another cause, corresponding to an HRS-acute kidney injury stage ≥1.^24^ Response to volume expansion was not included as part of HRS-criteria.

### Collection of stool samples

One stool sample and fecal swab from each participant was collected within the first week of hospitalization.

The stool sample of approximately 5 g of fecal material was immediately mixed with 10 mL RNAlater. All samples were transferred to Copenhagen University Hospital Hvidovre and immediately stored at −80°C until further processing. Samples were stored for up to 12 months until DNA extraction was performed. All samples were then transferred to Copenhagen University, Department of Plant and Environmental Sciences for further analyses. Fecal swabs collected from the stool samples were placed into Copan Liquid Amies Elution Swab (eSwab®) transport tubes (Copan, Italy) and stored at 5°C and analyzed within 48 h.

### Culturing of fecal swabs, semi-quantification, and identification

At Copenhagen University Hospital Hvidovre’s Department of Clinical Microbiology each fecal swab was shaken for 5 s and 10 μl was inoculated onto agar plates, including a bi-plate agar (5% horse blood and chromogenic agar) and CHROMagar™ StrepB. The agar plates were incubated at 35°C in ambient air for 24 h. The growth rate of bacteria was estimated semi-quantitatively as 0 (no growth), 1 × 10^2^, 1 × 10^3^, 1 × 10^4^, or ≥1 × 10^5^ CFU/ml. Bacteria were identified using Microflex matrix-assisted laser desorption ionization time-of-flight (MALDI-TOF) mass spectrometry (Bruker Daltonics, Billerica, MA, USA) with FlexAnalysis™ software. Accurate identification required scores of ≥2.0 for the species level and ≥1.7 for the genus level.^25^ If *E. faecalis* was found, the strain was frozen in 10% glycerol and kept at −80°C for DNA extraction and PCR analysis.

### DNA extraction

DNA extraction was performed at Copenhagen University Hospital Hvidovre’s Department of Clinical Microbiology. In total, microbial DNA was extracted from 30 stool samples dissolved in RNAlater; seven *E. faecalis* strains were identified from stool samples, and four known bacterial strains: two cytolysin-positive controls (*E. faecalis* ATCC 51,299 and *E. faecalis* ATCC 29,212) and two cytolysin-negative controls (*Escherichia coli* ATCC 25,922 and *Staphylococcus aureus* ATCC 29,213). The DNA extraction kit used for all samples and bacterial strains was the DNeasy Powersoil Pro Kit (Qiagen Inc., USA).

For stool samples, fecal material corresponding to the loop size of a 10 µL inoculation loop was transferred to a Powerbead Pro tube with 800 µL CD1 buffer and the protocol was followed according to the manufacturer’s instructions.

*E. faecalis* isolates from study participants and positive control isolates on 5% blood agar plates were inoculated in 1.3 mL serum bouillon and incubated overnight at 37°C in a thermoshaker set at 250 rpm. From each overnight culture, 800 µL was transferred to a Powerbead Pro tube and the protocol was followed according to the manufacturer’s instructions.

DNA yield was measured by fluorometric quantification with a Qubit 2.0 fluorometer (Invitrogen™, Life Technologies, CA 92,008, USA) using the Qubit 1X dsDNA HS Assay Kit (Invitrogen, cat. nr. Q33231, Eugene, Oregon, USA).

### Quantitative PCR

Quantitative polymerase chain reaction (qPCR) procedures were performed at Copenhagen University’s Department of Plant and Environmental Sciences. A reaction mix was made on ice, consisting of Sigma H_2_O 5.4 µL/sample, BSA (20 mg/µL) 1 µL/sample, Forward primer 10 µM 0.8 µL/sample, and Reverse primer 10 µM 0.8 µL/sample. The primers used were cylL_S__R + cylL_L__F (see Supplementary Table S1 for primer sequences).

When analyzing samples, 2.5 µL eluted DNA was used; for negative controls, 2 µL Sigma H_2_O was added. All the samples were preheated to 50°C for 2 min. Thereafter, 10 µL Brilliant III Ultra-Fast SYBR Green Low ROX qPCR Master Mix (Agilent Technologies, United States) (also preheated to 50°C) was added, and the samples were mixed. qPCR was performed using the AriaMx PCR System (Agilent Technologies, United States) and the following terminal program: initial denaturation at 95°C for 3 min, followed by 40 cycles of 95°C denaturing for 5 sec and annealing for 40 sec at 62.2°C. This was followed by a melting curve from 55°C to 95°C with 0.5°C resolution and holding the temperature at each step for 5 sec. PCR products were visualized using agarose gel electrophoresis stained with GelRed (Biotium, United States), to inspect for unspecific amplification.

### In silico PCR and database search

*In silico* PCR was performed using CLC genomic Workbench V22 (QIAGEN) and Primer blast.^26^ The *E. faecalis* strains used had already been sequenced and are available under the BioSample ID SAMEA114334018-SAMEA114334027. Sequences from *E. faecalis* strains from Duan et al. 2019,^17^ used as references, can be found in the European Nucleotide Archive (ENA) under accession number PRJEB25007.

To investigate the genomic organization of the cylL_S_ and cyl-_L_ genes, we performed a BLASTN search, limited to records that included *Enterococcus faecalis* (taxid:1351), using the complete nucleotide sequence of cylL_S_ and cylL_L_ genes, including the sequence between the two genes, from our cytolysin-positive control strain (ATCC51299) as a query against the BLAST nucleotide (nt/nr) database (carried out in December 2024).

### Primer selection

We performed an *in silico* PCR on 10 Danish *E. faecalis* strains isolated in a previous study (bio sample ID SAMEA114334018-SAMEA114334027), in order to ensure that the primers used previously to substantiate the correlation between cytolysin and mortality in patients suffering from AH^17^would also be able to identify cytolysin genes in a different set of Danish strains of *E. faecalis*. Of these 10 strains, two encoded the cytolysin genes cylL_L_ and cylL_S_. The *in silico* PCR revealed that both Danish isolates contained the same 3 bp mismatch in the cylL_S__F primer (see Supplementary Table S1). Upon further investigation, we discovered that these mismatches were also present in the strains isolated in the study by Duan et al. in 2019.^17^ Furthermore, the first use of the cylLS_F primer by Shepard et al. in 2002, as well as the first sequence of the cylLS and cylLL genes that the primers are based on, contains a different sequence with no mismatches.^27,28^ Therefore, the mismatches appear to be an error in the primer sequence and not a mutation in the bacterial DNA. Although the mismatches are in the 5’ end of the primer, and were thus unlikely to prevent the PCR reaction, we decided against duplicating the mismatches and did not use the cylLS_F primer.

CylL_S_ and cylL_L_ are two subunits that are both required for the formation of the final toxin.^28,29^ In the reference strains and the Danish isolates, the genes encoding the CylL_S_ and CylL_L_ peptides are found next to each other in the same operon. This allowed us to use the forward primer from the cylL_L_ primer set and the reverse primer from the cylL_S_ primer set (see Supplementary Table S1 for the primer sequence), in order to simultaneously test for the presence of both cytolysin genes without using the erroneous primer. This had the added advantage of producing a longer PCR product (201 bp compared to 52 and 65 bp for cylL_L_ and cylL_S_, respectively) that is easier to separate from potential primer-dimers by gel electrophoresis. To ensure that a large fraction of cytolysin positive *E. faecalis* did not have a different genome organization of the cylL_S_ and cylL_L_ genes that could prevent the longer PCR reaction we investigated the organization of the cylL_S_ and cylL_L_ genes in publicly available genomes using BLASTN. The BLASTN search returned 205 hits, of which only five sequences did not share the same gene organization, having less than 99% coverage of the query sequence. Three of the hits were for incomplete sequences where only part of the cytolysin operon was sequenced (accession numbers AF394225.1, OR405531.1, and OR405532.1). The remaining two hits were from strains that contained only the cylL_S_ gene, but not the cylL_L_. These two strains were uploaded by the same author but annotated as being from different samples, albeit both from South Korea (accession numbers CP136353.1 and CP138642.1). Using the longer PCR product, we were therefore able to reliably test for the presence of both genes simultaneously.

### Cytolysin positivity

Samples were defined as cytolysin-positive if they i) had a band in the gel electrophoresis of the expected size (201 bp), ii) had a low CT value (< 30), and iii) had a melting curve that matched that of the positive control. The same parameters were used to evaluate cytolysin positivity in DNA from *E. faecalis* strains isolated from patient fecal samples, as well as in positive and negative control strains.

### Study endpoints

The primary endpoint was a comparison of the 180-d mortality in AH patients with cytolysin-positive and -negative fecal samples. The secondary endpoints were comparisons of the 30-, 90- and 365-d mortality rates in AH patients with and without cytolysin-positive fecal samples.

The exploratory outcomes were differences in baseline characteristics in AH patients with cytolysin-positive and -negative fecal samples, and differences in lengths of hospital stay, treatments received for AH, and complications of liver disease.

### Sample size

Power calculations were made according to the following assumptions, themselves based on the results reported by Duan et al.:^17^ an expected mortality of 75% in patients with cytolysin-positive fecal samples, an expected mortality of 10% in patients with cytolysin-negative fecal samples, and a predicted 30% of patients with AH to have cytolysin-positive stool samples. Based on power calculations carried out using a significance level of 0.05 and a power above 0.8, at least 20 patients with alcohol-associated hepatitis was needed. Power calculations were performed using https://clincalc.com/stats/samplesize.aspx. We planned to include a total of 30 participants in the trial group to further increase our statistical power to detect significant differences between the groups.

### Statistical analysis

Baseline differences in mean age, GAHS, Child-Pugh Score, CCI, MELD, bilirubin, INR, albumin, creatinine, platelets, and ALT between individuals with cytolysin-positive and cytolysin-negative fecal samples were compared using a two-sample *t*-test. If the data were not normally distributed, the Mann–Whitney U Test was used.^30^ We also calculated the mean ABIC score^31^ and Maddrey’s discriminant function index (DFI)^32^ using the same statistical analyses. Fisher’s exact test was used to compare baseline differences in gender ratios, previous AH and cirrhosis, and differences in the proportions of complications of liver disease, treatment with corticosteroids or antibiotics, and the risk of re-admission between individuals with cytolysin-positive and cytolysin-negative fecal samples. Fisher’s exact test was also used to calculate the difference (in proportion) of survival after 30, 90, 180 and 365 d between individuals with cytolysin-positive and cytolysin-negative fecal samples. Log-rank test was used to calculate differences in survival between groups throughout the first year. *P*-values lower than 0.05 were considered statistically significant. All calculations were performed in R Statistics version 2024.09.0 + 375.

### Ethics

The study was approved by the Ethics Committee of the capital region of Denmark (H-21041462) and conducted in accordance with the revised Declaration of Helsinki. The study was registered at clinicaltrials.gov (NCT05618418). All participants were given verbal and written information and provided written informed consent for their participation in the study.

### Role of the funding source

The funder of the study had no role in study design, data collection, data analysis, data interpretation, or writing of the report.

## Results

**Figure 1.**
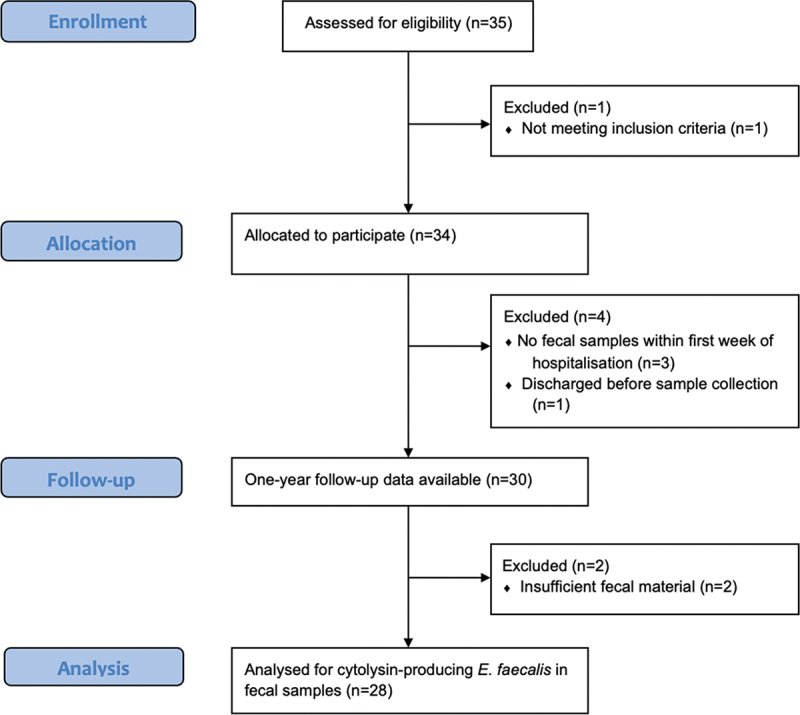
Flow diagram for recruiting patients. n, number; E., *enterococcus.*

Thirty-four patients with AH were invited to participate in the BATTLE trial (Fig. 1); all accepted. Following inclusion, four patients were excluded because no fecal samples were collected within the first week of hospitalization (*n* = 3) or because of discharge before a sample was collected (*n* = 1). The median time of sampling was 4 d following hospital admission (range 2–7 d). Following fecal analysis, 2 of the 30 patients were excluded from further analyses due to insufficient fecal material.

### Cytolysin-positive E. faecalis

Three patients (10.7%) were identified as cytolysin-positive based on qPCR and the criteria described above. We successfully isolated *E. faecalis* strains from seven patients (39%). Of these seven strains, three were cytolysin-positive, as confirmed by qPCR. The three strains originated from the same three patients identified as having cytolysin-positive fecal samples. There were no significant differences at baseline between cytolysin-positive and -negative patients in age, gender distribution, previous AH, cirrhosis, liver disease scores, or biochemical variables (Table 1). However, we observed a higher Child–Pugh score in the three cytolysin-positive patients although this finding was not statistically significant.

**Table 1.** Patient demographics and baseline characteristics.

	Cytolysin- positive (*N*=3)	Cytolysin-negative (*N*=25)	*P*-value for difference
Mean (SD) age, year	59.3 (11)	57.3 (11.5)	0.88
Male, n (%)	2 (66.6)	18 (72)	1
Previous alcohol-associated hepatitis, n (%)	0 (0)	2 (8)	1
Cirrhosis, n (%)^a^	3 (100)	17 (68)	0.59
Type 2 diabetes, n (%)	1 (33)	2 (8)	0.37
Mean (SD) Child-Pugh score	13 (1.4)	9.1 (2.0)	0.08
Mean (SD) CCI	5.3 (3.8)	2.4 (1.9)	0.32
Mean (SD) GAHS	9 (2)	8.4 (1.2)	0.66
Severe alcohol-associated hepatitis, n (%)	2 (66)	14 (56)	1
Mean (SD) MELD	18.6 (9.2)	18.8 (6.7)	0.97
Mean (SD) Maddrey’s DF	65.0 (32.8)	58.6 (39.6)	0.62
Maddrey’s DF > 32, n (%)	3 (100)	22 (88)	1
Mean (SD) ABIC	8.6 (1.3)	8.6 (0.8)	0.94
Mean (SD) bilirubin μmol/L	183.3 (91.7)	219.8 (143.9)	0.82
Mean (SD) INR	1.87 (0.6)	1.94 (0.6)	0.6
Mean (SD) albumin g/L	21 (1)	21.8 (6)	0.57
Mean (SD) creatinine μmol/L	80.3 (36.8)	77.5 (37.7)	0.88
Mean (SD) platelets 10^9^/L	119 (139.2)	137 (74.5)	0.85
Mean (SD) ALT U/L	46.3 (35.6)	67.1 (42.4)	0.46

ABIC, age, serum bilirubin, INR, serum-creatinine; ALT, alanin-aminotransferase; CCI, Charlson Comorbidity Index; DF, discriminant function; GAHS, Glasgow Alcoholic Hepatitis Score; INR, international normalised ratio; MELD, Model for End-Stage Liver Disease; SD, standard deviation.

^a^Data missing for one patient in the cytolysin-negative group.

### Mortality

**Figure 2.**
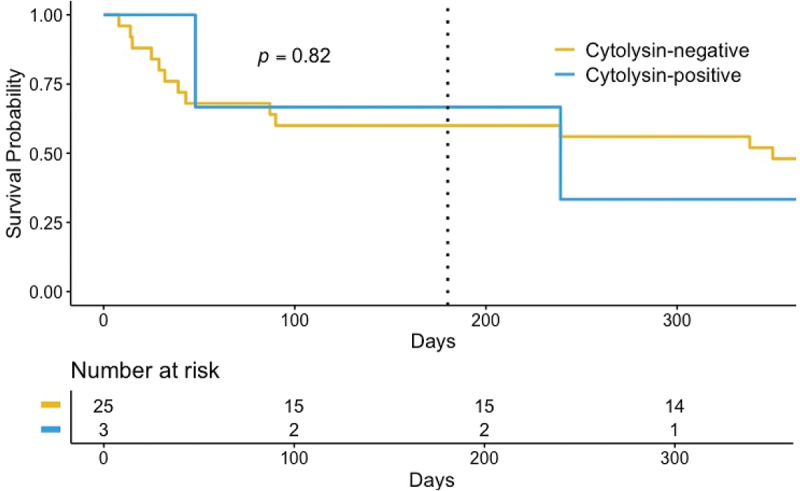
Survival probability of patients with alcohol-associated hepatitis with cytolysin-positive and cytolysin-negative fecal samples.

In the year following their primary hospital admission, 15 of the 28 (53.6%) patients with AH had died; we found no difference between the cytolysin-positive and cytolysin-negative groups (log-rank test for difference between groups, *p* = 0.82). At the primary endpoint of 180 d following hospital admission, 10 of the 28 (36%) patients with AH had died (Fig. 2): one of the three (33%) patients with cytolysin-positive *E. faecalis*, and nine of the 25 (36%) patients with cytolysin-negative *E. faecalis*, with no significant difference between the groups (*p* = 1.0) (Table 2). There was no significant difference in mortality between patients with cytolysin-positive and cytolysin-negative fecal samples at the pre-specified timepoints of 30, 90, and 365 d following hospital admission.

**Table 2.** Treatment of alcohol-associated hepatitis and complications of liver disease following initial hospital admission.

	Cytolysin- positive *E. faecalis* (*n*=3)	Cytolysin- negative *E. faecalis* (*n*=25)	*P*-value for difference
Mean (SD) number of days of hospitalization	14.3 (4.7)	14.0 (7.3)	0.65
Treatment with corticosteroids, n (%)	0 (0)	10 (40)	0.53
Treatment with antibiotics, n (%)	0 (0)	16 (64)	0.06
Complications of liver disease:			
HE, n (%)	1 (33.3)	17 (68)	0.28
Ascites, n (%)	2 (66.7)	16 (64)	1
Variceal bleeding, n (%)	0 (0)	1 (4)	1
HRS, n (%)	1 (33.3)	7 (28)	1
ICU, n (%)	0 (0)	1 (4)	1
Patient dying during initial hospital admission, n (%)	0 (0)	4 (16)	1
Mortality			
30 days 90 days 180 days 365 days	0 (0) 1 (33.3) 1 (33.3) 2 (66.7)	5 (20) 9 (36) 10 (33) 13 (52%)	1 1 1 1

ICU, Intensive Care Unit; HE, Hepatic Encephalopaty; HRS, Hepatorenal Syndrome; SD, standard deviation.

### Causes of death

Of the 15 patients that died within the first year of follow-up, four died during their primary hospitalization; all four died of causes related to liver failure (Supplementary Table S2). Of the 11 other patients dying after their primary hospitalization, eight died of complications of liver failure during a new hospitalization or shortly after discharge.

### Treatment and complications of liver disease

The mean length of the primary hospitalization was comparable between cytolysin-positive and cytolysin-negative patients. There was no significant difference between the groups in the number of patients treated with corticosteroids and antibiotics. None of the complications of liver disease differed significantly in frequency between the two groups.

In the patients receiving antibiotic treatment for various infections nine of the sixteen treated patients received treatment prior to fecal sampling (Suplementary Table S3). There was no significant difference in survival following 6 months between patients receiving antibiotic treatment during the initial hospital admission compared to patients not receiving antibiotic treatment (Fishers exact test for difference between groups, *p* = 0.25).

No patients had initiated antibiotic treatment or corticosteroids prior to hospital admission.

## Discussion

The results from this prospective, Danish cohort confirm the high mortality rate among hospitalized patients with AH that has been described in the literature.^8,35,36^ However, we were not able to reproduce the finding that cytolysin-positive *E. faecalis* correlates with increased mortality among hospitalized patients with AH, either at the primary outcome of 180 d or after 1 y following hospitalization. The incidence of cytolysin-positive *E. faecalis* that we found was also lower than that found by Duan et al.,^17^ where 25 of 79 (31.6%) patients were cytolysin-positive. In our cohort, only three of the 28 (10.7%) patients were cytolysin-positive. Furthermore, we did not detect any significant differences in baseline characteristics or other outcomes, such as complications of liver disease and the number of days of hospitalization, between AH patients with cytolysin-positive and cytolysin -negative fecal samples.

The strength of this study is that it is the first independent cohort to attempt to reproduce the findings reported by Duan et al. in 2019.^17^ The mortality rate of 36% that we observed in our cohort after 180 d is comparable to that reported by Duan et al. and to other cohorts of hospitalized AH patients.^5,17^

However, there are several limitations to this study. First, due to the sample size of only 28 patients with AH, only three of whom were cytolysin-positive, we cannot exclude the possibility that cytolysin-producing *E. faecalis* does, in fact, have an impact on mortality in hospitalized Danish patients with AH. Larger studies are needed to investigate this correlation.

There is also a risk that four of the 11 patients that was registered to die within the first 6 months following hospitalization (See Supplementary Table S2) were in fact presenting with end-stage alcohol-related liver disease in the form of cirrhosis with jaundice instead of alcohol-associated hepatitis.

Another limitation is some differences, albeit minor, in the handling of the fecal samples and analysis of cytolysin-positivity in our study compared to that of Duan et al.,^17^ in particular the primers (see “Primer selection”). However, we find it unlikely that the small differences in methodologies can explain the large differences between cytolysin-positivity and mortality observed between the two cohorts.

Potentially the chance of finding cytolysin-positive *E.* *faecalis* in fecal samples collected after initiation of corticosteroids or antibiotics prior to fecal sampling can be altered. The median time of sampling following hospitalization was identical to the time reported by Duan et al.^17^ In our cohort, however, no patients were receiving antibiotics or corticosteroids at hospital admission in comparison with 50% receiving antibiotics and 37.5% receiving corticosteroids reported by Duan et al. If these treatments can affect cytolysin positivity this potentially can explain some of differences in our reported findings.

The reasons we were not able to reproduce the findings reported by Duan et al. could be because of unknown differences between the two cohorts of AH patients. For instance, in Denmark fewer antibiotics are prescribed compared to the countries (USA, UK, Mexico, France, and Spain) from which patients were recruited in the study by Duan et al.^34,37^ Hence, the gut microbiome of Danish patients with AH (and of the Danish population generally) might have a different composition, including the less frequent presence of cytolysin-positive *E. faecalis*, than that of the aforementioned countries.

Our understanding of the role of the gut microbiome in many diseases, and the possibility to manipulate it to our advantage, has increased considerably in recent years.^38,39^ Changes in the gut microbiome are known to have an impact on the development of AH,^12,33^ which has led to several studies of gut microbiome-modulating treatments, so far with mixed results.^6,11,40^ The simplest way to manipulate the gut microbiome is by broad-spectrum antibiotic treatment, but results from studies of this topic have not, so far, shown a survival benefit in patients with AH. Støy et al. treated hospitalized patients with AH for 7 d with vancomycin 500 mg, gentamycin 40 mg, and meropenem 500 mg once daily, or matching placebos, and found no changes in markers of bacterial translocation, liver or systemic inflammation, or differences in survival.^15^ Louvet et al. treated 284 patients with severe AH for 30 d with prednisolone combined with amoxicillin-clavulanate, or prednisolone combined with placebo, and reported no difference in survival in the first 180 d following treatment.^41^

The transfer of a “full” gut microbiome from a healthy donor via FMT has also been investigated as a treatment for AH, with more promising results.^42^ In 2023, Pande et al.^6^ reported results from FMT treatment of 120 patients with severe AH who were randomized in an open-label study to prednisolone 40 mg/day for 28 d or healthy donor FMT through naso-duodenal tube daily for 7 d. The 90-d survival rate was significantly higher (*p* = 0.044) in the FMT group, at 75% (45/60), than in the prednisolone group, at 56.6% (34/60).

Another promising way of manipulating the gut microbiome is by bacteriophage therapy, where single bacterial strains can be targeted.^43,44^ Duan et al.^17^ were able to attenuate liver damage caused by alcohol in mice with cytolysin-producing *E. faecalis* by treating them with bacteriophages targeting this specific bacterial strain. If other research groups are able to reproduce a strong correlation between cytolysin-positive *E. faecalis* and mortality in patients with AH, bacteriophage therapy would offer an obvious treatment opportunity. However, bacteriophage therapy has mainly been reported from single cases as last resort treatment why the expectation to this treatment must be proved through randomized placebo-controlled trials before application to patients outside research settings.^45^ Furthermore, the lack of regulatory clarity with regards to bacteriophage therapy makes it difficult to envisage as part of routine treatment of AH within the foreseeable future.^46–48^

The limited treatment options for hospitalized patients with AH, and their high mortality, makes potential breakthroughs essential and is why we urge other researchers to continue to investigate whether certain bacterial strains, such as cytolysin-producing *E. faecalis*, correlate with mortality. In 2023, Cabre et al. published a methodological paper outlining the steps for analyzing cytolysin-positivity in fecal samples,^49^ and we hope this will help other researchers conduct similar comparisons in other cohorts of AH patients.

## Conclusion

In this cohort of hospitalized AH patients, we report a low incidence of cytolysin-positive *E. faecalis* and no increased mortality compared to AH patients without this bacterial strain. We urge researchers to continue to investigate the correlation between the gut microbiome and mortality in AH patients, in the hope of developing new treatments for the disease.

## Highlights

- Alcohol-associated hepatitis (AH) has a high long-term mortality with no approved treatments improving survival
- Cytolysin-producing *E. faecalis* has been reported as highly correlated to mortality
- We report a low incidence of Cytolysin-producing *E. faecalis* in Danish AH patients
- We report no correlation to mortality in Danish hospitalized AH patients

## Supplementary Material

130825Supplementary.docx

## Data Availability

The data that support the findings of this study are not openly available due to reasons of sensitivity, due to the General Data Protection Regulation of the European Union, but anonymized data are available from the corresponding authors upon reasonable request.

## Clinical trial number

NCT05618418.

## References

[1] Bataller R, Arab JP, Shah VH, Hardin CC. Alcohol-associated hepatitis. N Engl J Med. 2022;387(26):2436–12. doi: 10.1056/NEJMra2207599. 36577100

[2] Kasper P, Lang S, Steffen H, Demir M. Management of alcoholic hepatitis: a clinical perspective. Liver Int. 2023;43(10):2078–2095. doi: 10.1111/liv.15701. 37605624

[3] Stickel F, Moreno C, Hampe J, Morgan MY. The genetics of alcohol dependence and alcohol-related liver disease. J Hepatol. 2017;66(1):195–211. doi: 10.1016/j.jhep.2016.08.011. 27575312

[4] Ventura-Cots M, Argemi J, Jones PD, Lackner C, El Hag M, Abraldes JG, Alvarado E, Clemente A, Ravi S, Alves A, et al. Clinical, histological and molecular profiling of different stages of alcohol-related liver disease. Gut. 2022;71(9):1856–1866. doi: 10.1136/gutjnl-2021-324295. 34992134 PMC11034788

[5] Arab JP, Díaz LA, Baeza N, Idalsoaga F, Fuentes-López E, Arnold J, Ramírez CA, Morales-Arraez D, Ventura-Cots M, Alvarado-Tapias E, et al. Identification of optimal therapeutic window for steroid use in severe alcohol-associated hepatitis: a worldwide study. J Hepatol. 2021;75(5):1026–1033. doi: 10.1016/j.jhep.2021.06.019. 34166722 PMC11090180

[6] Pande A, Sharma S, Khillan V, Rastogi A, Arora V, Shasthry SM, Vijayaraghavan R, Jagdish R, Kumar M, Kumar G, et al. Fecal microbiota transplantation compared with prednisolone in severe alcoholic hepatitis patients: a randomized trial. Hepatol Int. 2023;17(1):249–261. doi: 10.1007/s12072-022-10438-0. 36469298

[7] Louvet A, Thursz MR, Kim DJ, Labreuche J, Atkinson SR, Sidhu SS, O’Grady JG, Akriviadis E, Sinakos E, Carithers RL, et al. Corticosteroids reduce risk of death within 28 days for patients with severe alcoholic hepatitis, compared with pentoxifylline or placebo—a meta-analysis of individual data from controlled trials. Gastroenterology. 2018;155(2):458–468.e8. doi: 10.1053/j.gastro.2018.05.011. 29738698

[8] Jophlin LL, Singal AK, Bataller R, Wong RJ, Sauer BG, Terrault NA, Shah VH. Acg clinical guideline: alcohol-associated liver disease. Am J Gastroenterol. 2024;119(1):30–54. doi: 10.14309/ajg.0000000000002572. 38174913 PMC11040545

[9] Thursz MR, Richardson P, Allison M, Austin A, Bowers M, Day CP, Downs N, Gleeson D, MacGilchrist A, Grant A, et al. Prednisolone or pentoxifylline for alcoholic hepatitis. N Engl J Med. 2015;372(17):1619–1628. doi: 10.1056/NEJMoa1412278. 25901427

[10] Zhu L, Wang Y, Pan CQ, Xing H. Gut microbiota in alcohol-related liver disease: pathophysiology and gut-brain cross talk. Front Pharmacol. 2023;14:1258062. doi: 10.3389/fphar.2023.1258062. 37601074 PMC10436520

[11] Sarin SK, Pande A, Schnabl B. Microbiome as a therapeutic target in alcohol-related liver disease. J Hepatol. 2019;70(2):260–272. doi: 10.1016/j.jhep.2018.10.019. 30658727

[12] Fairfield B, Schnabl B. Gut dysbiosis as a driver in alcohol-induced liver injury. JHEP Rep. 2021;3(2):100220. doi: 10.1016/j.jhepr.2020.100220. 33598648 PMC7868813

[13] Israelsen M, Alvarez-Silva C, Madsen BS, Hansen CD, Torp NC, Johansen S, Hansen JK, Prier Lindvig K, Insonere J, Riviere V, et al. Impact of acute alcohol consumption on circulating microbiome in asymptomatic alcohol-related liver disease. Gut. 2024;73(6):1041–1044. doi: 10.1136/gutjnl-2023-330360. 37344168

[14] Hsu CL, Wang Y, Duan Y, Chu H, Hartmann P, Llorente C, Zhou R, Schnabl B. Differences in bacterial translocation and liver injury in ethanol versus diet-induced liver disease. Dig Dis Sci. 2023;68(7):3059–3069. doi: 10.1007/s10620-023-07860-1. 36807831 PMC10313731

[15] Støy S, Laursen TL, Eriksen LL, Grønbæk H, Vilstrup H, Sandahl TD. No effect in alcoholic hepatitis of gut-selective, broad-spectrum antibiotics on bacterial translocation or hepatic and systemic inflammation. Clin Transl Gastroenterol. 2021;12(2):e00306. doi: 10.14309/ctg.0000000000000306. 33566559 PMC7846454

[16] Bajaj JS, Gavis EA, Fagan A, Wade JB, Thacker LR, Fuchs M, Patel S, Davis B, Meador J, Puri P, et al. A randomized clinical trial of fecal microbiota transplant for alcohol use disorder. Hepatology. 2021;73(5):1688–1700. doi: 10.1002/hep.31496. 32750174

[17] Duan Y, Llorente C, Lang S, Brandl K, Chu H, Jiang L, White RC, Clarke TH, Nguyen K, Torralba M, et al. Bacteriophage targeting of gut bacterium attenuates alcoholic liver disease. Nature. 2019;575(7783):505–511. doi: 10.1038/s41586-019-1742-x. 31723265 PMC6872939

[18] Forrest EH. Analysis of factors predictive of mortality in alcoholic hepatitis and derivation and validation of the Glasgow alcoholic hepatitis score. Gut. 2005;54(8):1174–1179. doi: 10.1136/gut.2004.050781. 16009691 PMC1774903

[19] Pugh RN, Murray-Lyon IM, Dawson JL, Pietroni MC, Williams R. Transection of the oesophagus for bleeding oesophageal varices. J Educ Chang British Surgery. 1973;60(8):646–649. doi: 10.1002/bjs.1800600817. 4541913

[20] Charlson ME, Pompei P, Ales KL, MacKenzie CR. A new method of classifying prognostic comorbidity in longitudinal studies: development and validation. J Chronic Dis. 1987;40(5):373–383. doi: 10.1016/0021-9681(87)90171-8. 3558716

[21] Kamath PS, Kim RW. The model for end-stage liver disease (MELD). Hepatology. 2007 45. 45(3):797–805. doi: 10.1002/hep.21563. 17326206

[22] Thursz M, Gual A, Lackner C, Mathurin P, Moreno C, Spahr L, Sterneck M, Cortez-Pinto H. EASL clinical practice guidelines: Management of alcohol-related liver disease. J Hepatol. 2018;69(1):154–181. doi: 10.1016/j.jhep.2018.03.018. 29628280

[23] Louvet A, Naveau S, Abdelnour M, Ramond M-J, Diaz E, Fartoux L, Dharancy S, Texier F, Hollebecque A, Serfaty L, et al. The Lille model: a new tool for therapeutic strategy in patients with severe alcoholic hepatitis treated with steroids. Hepatology. 2007;45(6):1348–1354. doi: 10.1002/hep.21607. 17518367

[24] Angeli P, Gines P, Wong F, Bernardi M, Boyer TD, Gerbes A, Moreau R, Jalan R, Sarin SK, Piano S, et al. Diagnosis and management of acute kidney injury in patients with cirrhosis: revised consensus recommendations of the International Club of Ascites. Gut. 2015;64(4):531–537. doi: 10.1136/gutjnl-2014-308874. 25631669

[25] Khot PD, Couturier MR, Wilson A, Croft A, Fisher MA. Optimization of matrix-assisted laser desorption ionization–time of flight mass spectrometry analysis for bacterial identification. J Clin Microbiol. 2012;50(12):3845–3852. doi: 10.1128/JCM.00626-12. 22993178 PMC3502975

[26] Ye J, Coulouris G, Zaretskaya I, Cutcutache I, Rozen S, Madden TL. Primer-BLAST: a tool to design target-specific primers for polymerase chain reaction. BMC Bioinf. 2012;13(1):134. doi: 10.1186/1471-2105-13-134. PMC3412702 22708584

[27] Shepard BD, Gilmore MS. Differential expression of virulence-related genes in Enterococcus faecalis in response to biological cues in serum and urine. Infect Immun. 2002;70(8):4344–4352. doi: 10.1128/IAI.70.8.4344-4352.2002. 12117944 PMC128128

[28] Gilmore MS, Segarra RA, Booth MC, Bogie CP, Hall LR, Clewell DB. Genetic structure of the Enterococcus faecalis plasmid pAD1-encoded cytolytic toxin system and its relationship to lantibiotic determinants. J Bacteriol. 1994;176(23):7335–7344. doi: 10.1128/jb.176.23.7335-7344.1994. 7961506 PMC197123

[29] Booth MC, Bogie CP, Sahl H, Siezen RJ, Hatter KL, Gilmore MS. Structural analysis and proteolytic activation of Enterococcus faecalis cytolysin, a novel lantibiotic. Mol Microbiol. 1996;21(6):1175–1184. doi: 10.1046/j.1365-2958.1996.831449.x. 8898386

[30] Mann–Whitney U Test. Encyclopedia of research design, 2455 Teller Road, Thousand Oaks, California 91320. United States: SAGE Publications, Inc.; 2010. doi: 10.4135/9781412961288.n228.

[31] Dominguez M, Rincón D, Abraldes JG, Miquel R, Colmenero J, Bellot P, García-Pagán J-C, Fernández R, Moreno M, Bañares R, et al. A new scoring system for prognostic stratification of patients with alcoholic hepatitis. Am J Gastroenterol. 2008;103(11):2747–2756. doi: 10.1111/j.1572-0241.2008.02104.x. 18721242

[32] Maddrey WC, Boitnott JK, Bedine MS, Weber FL, Mezey E, White RI. Corticosteroid therapy of alcoholic hepatitis. Gastroenterology. 1978;75(2):193–199. doi: 10.1016/0016-5085(78)90401-8. 352788

[33] Smirnova E, Puri P, Muthiah MD, Daitya K, Brown R, Chalasani N, Liangpunsakul S, Shah VH, Gelow K, Siddiqui MS, et al. Fecal microbiome distinguishes alcohol consumption from alcoholic hepatitis but does not discriminate disease severity. Hepatology. 2020;72(1):271–286. doi: 10.1002/hep.31178. 32056227 PMC7752764

[34] Browne AJ, Chipeta MG, Haines-Woodhouse G, Kumaran EPA, Hamadani BHK, Zaraa S, Henry NJ, Deshpande A, Reiner RC, Day NPJ, et al. Global antibiotic consumption and usage in humans, 2000–18: a spatial modelling study. The Lancet Planet Health. 2021;5(12):e893–904. doi: 10.1016/S2542-5196(21)00280-1. 34774223 PMC8654683

[35] Philips CA. A comprehensive review of diagnosis and management of alcohol-associated hepatitis. Sage Open Med. 2024;12:12. doi: 10.1177/20503121241297000. PMC11549690 39526098

[36] Hazrat KG, Støy SH, Sandahl TD, Jepsen P. Incidence and mortality of alcohol-related hepatitis in Denmark – an update, 2016–2023. JHEP Rep. 2025;7(6):101390. doi: 10.1016/j.jhepr.2025.101390. 40486135 PMC12143810

[37] Malo-Fumanal S, Rabanaque-Hernández MJ, Feja-Solana C, Lallana-Alvarez MJ, Armesto-Gómez J, Bjerrum L. Differences in outpatient antibiotic use between a Spanish region and a Nordic country. Enferm Infecc Microbiol Clin. 2014;32(7):412–417. doi: 10.1016/j.eimc.2013.10.002. 24262316

[38] Schmartz GP, Rehner J, Gund MP, Keller V, Molano L-A, Rupf S, Hannig M, Berger T, Flockerzi E, Seitz B, et al. Decoding the diagnostic and therapeutic potential of microbiota using pan-body pan-disease microbiomics. Nat Commun. 2024;15(1):8261. doi: 10.1038/s41467-024-52598-7. 39327438 PMC11427559

[39] Hou K, Wu Z-X, Chen X-Y, Wang J-Q, Zhang D, Xiao C, Zhu D, Koya JB, Wei L, Li J, et al. Microbiota in health and diseases. Sig Transduct Target Ther. 2022;7(1):135. doi: 10.1038/s41392-022-00974-4. PMC9034083 35461318

[40] Lang S, Fairfied B, Gao B, Duan Y, Zhang X, Fouts DE, Schnabl B. Changes in the fecal bacterial microbiota associated with disease severity in alcoholic hepatitis patients. Gut Microbes. 2020;12(1):1785251. doi: 10.1080/19490976.2020.1785251. 32684075 PMC7524371

[41] Louvet A, Labreuche J, Dao T, Thévenot T, Oberti F, Bureau C, Paupard T, Nguyen-Khac E, Minello A, Bernard-Chabert B, et al. Effect of prophylactic antibiotics on mortality in severe alcohol-related hepatitis. JAMA. 2023;329(18):1558. doi: 10.1001/jama.2023.4902. 37159035 PMC10170332

[42] Taha AM, Abouelmagd K, Nada SA, Mahmoud AM, Nguyen D, Sharma S, Elewa M. Impact of fecal microbiota transplantation in severe alcoholic hepatitis: a systematic review and meta‐analysis. JGH Open. 2024 8. 8(8). doi: 10.1002/jgh3.70007. PMC11331245 39161797

[43] Fujiki J, Schnabl B. Phage therapy: targeting intestinal bacterial microbiota for the treatment of liver diseases. JHEP Rep. 2023;5(12):100909. doi: 10.1016/j.jhepr.2023.100909. 37965159 PMC10641246

[44] Pirnay J-P, Djebara S, Steurs G, Griselain J, Cochez C, De Soir S, Glonti T, Spiessens A, Vanden Berghe E, Green S, et al. Personalized bacteriophage therapy outcomes for 100 consecutive cases: a multicentre, multinational, retrospective observational study. Nat Microbiol. 2024;9(6):1434–1453. doi: 10.1038/s41564-024-01705-x. 38834776 PMC11153159

[45] Advocating for phage therapy. Nat Microbiol. 2024;9(6):1397–1398. doi: 10.1038/s41564-024-01733-7. 38839974

[46] Yang Q, Le S, Zhu T, Wu N. Regulations of phage therapy across the world. Front Microbiol. 2023;14. doi: 10.3389/fmicb.2023.1250848. PMC10588630 37869667

[47] Faltus T. The medicinal phage—regulatory roadmap for phage therapy under EU pharmaceutical legislation. Viruses. 2024;16(3):443. doi: 10.3390/v16030443. 38543808 PMC10974108

[48] Pirnay J-P, Ferry T, Resch G. Recent progress toward the implementation of phage therapy in Western medicine. FEMS Microbiol Rev. 2022;46(1):46. doi: 10.1093/femsre/fuab040. 34289033

[49] Cabré N, Yang Y, Wang Y, Schnabl B. Development of a quantitative PCR method for detecting Enterococcus faecalis cytolysin in human stool samples. MPs. 2023;6(6):107. doi: 10.3390/mps6060107. 37987354 PMC10660514

