# Supplementary material for: Low incidence of cytolysin-positive E. faecalis and no correlation to survival in Danish patients with alcohol-associated hepatitis: A prospective cohort study
Source: Gut Microbes Rep. 2025 Sep 3;2(1):2549729. doi: 10.1080/29933935.2025.2549729 (PMC12940113; doi:10.1080/29933935.2025.2549729)
Supplement: 130825Supplementary.docx [file KGMR_A_2549729_SM7694.docx]

**Appendix**

**Supplementary Table 1. Primers described in the study.**

| Primer name | Sequence | Reference |
| --- | --- | --- |
| Primers used in the study: | | |
| cylL_S__R | 5'-CAAAAGAAGGACCAACAAGTTCTAATT-3' | A,B,C,D&E |
| cylL_L__F | 5'-CTGTTGCGGCGACAGCT-3' | A,B,C&D |
| Primers described but not used in the study: | | |
| cylL_S__F incorrect | 5'-GTAAAATAAGTAAAATCAAGAAAACTATTACTC-3' | A,B&E |
| cylL_S__F correct* | 5'-GCTAAATAAGGAAAATCAAGAAAACTATTACTC-3' | C&D |
| Difference | 5'-.CT.......G......................-3' | This study |

Supplementary Table 1: Sequence of the primer sequences used in this study, as well as the primer sequence found to be incorrect and therefore not used. The incorrect primer sequence was used in several publications (references A, B, E). The original primer sequence was first described in Shepard and Gilmore 2002 (ref. C) and is based on the sequence of the CylL_S_ gene (ref. D). The correct primer is also a 100% match to the sequence of all cytolysin-positive *E. faecalis* strains tested (see ‘Primer selection’). * The ‘correct’ primer is here defined as the sequence that matches the genome sequence of available cytolysin positive *E. faecalis* genomes.

References for the Supplementary materials (not included in the list of References):

A: Duan Y, Llorente C, Lang S, Brandl K, Chu H, Jiang L, et al. Bacteriophage targeting of gut bacterium attenuates alcoholic liver disease. Nature 2019;575:505–11. https://doi.org/10.1038/s41586-019-1742-x.

B: Haas W, Shepard BD, Gilmore MS. Two-component regulator of Enterococcus faecalis cytolysin responds to quorum-sensing autoinduction. Nature 2002;415:84–7. https://doi.org/10.1038/415084a.

C: Shepard BD, Gilmore MS. Differential Expression of Virulence-Related Genes in Enterococcus faecalis in Response to Biological Cues in Serum and Urine. Infect Immun 2002;70:4344–52. https://doi.org/10.1128/IAI.70.8.4344-4352.2002.

D: Gilmore MS, Segarra RA, Booth MC, Bogie CP, Hall LR, Clewell DB. Genetic structure of the Enterococcus faecalis plasmid pAD1-encoded cytolytic toxin system and its relationship to lantibiotic determinants. J Bacteriol 1994;176:7335–44. https://doi.org/10.1128/jb.176.23.7335-7344.1994.

E: Cabré N, Yang Y, Wang Y, Schnabl B. Development of a Quantitative PCR Method for Detecting Enterococcus faecalis Cytolysin in Human Stool Samples. Methods Protoc 2023;6:107. https://doi.org/10.3390/mps6060107.

**Supplementary Table 2. Causes of death.**

| Cytolysin (Yes/No) | Death occurring during initial hospitalization | Time to death (days following hospitalization) | Cause of death |
| --- | --- | --- | --- |
| Yes | No | 239 | Terminal cirrhosis and HE during a new episode of AH |
| Yes | No | 48 | Terminal cirrhosis and HE during hospitalization following hip fracture |
| No | No | 338 | Unknown |
| No | No | 350 | Terminal cirrhosis and HE |
| No | Yes | 39 | HE and respiratory insufficiency |
| No | No | 90 | Demanded to be discharged without follow-up and died soon after |
| No | Yes | 14 | HRS and HE |
| No | No | 239 | HE, variceal bleeding, and respiratory insufficiency |
| No | No | 43 | Terminal cirrhosis (died shortly after discharge) |
| No | No | 25 | New diagnosis of C. oesophagus. Discharged to palliative care |
| No | No | 87 | Terminal cirrhosis and HE |
| No | No | 32 | Discharged to palliative care due to terminal cirrhosis |
| No | Yes | 8 | Death from complications of AH and terminal cirrhosis (variceal bleeding and HE) |
| No | Yes | 15 | HRS, HE |
| No | No | 29 | Terminal liver failure. Discharged to palliative care |

AH, alcohol-associated hepatitis; HE, hepatic encephalopathy; HRS, hepatorenal syndrome

**Supplementary Table 3. Antibiotic treatment under primary hospitalization**

| Type of Infection | Type of antibiotics (days of treatment following hospitalization) | Survival, days |
| --- | --- | --- |
| SBP | Piperacillin/Tazobactam i.v. (3-17)* | 338 |
| UTI | Selexid p.o. (3-5)* | 350 |
| *C. difficile* infection | Vancomycin p.o. (2-14)* | Alive following 365 days |
| Infection without focus | Piperacillin/Tazobactam i.v. (2-7)* | Alive following 365 days |
| SBP,  *E. faecium* in ascites | Piperacillin/Tazobactam i.v. (2-4)*, Metronidazole i.v. (2-4) and Vancomycin i.v. (4-10) | Alive following 365 days |
| Infection without focus | Piperacillin/Tazobactam i.v. (15) | Alive following 365 days |
| Pneumonia, *S. aureus* in blood | Piperacillin/Tazobactam i.v. (19-24) and Meropenem i.v. (25-38) | 39 |
| Infection without focus | Piperacillin/Tazobactam i.v. (2-13)* and Metronidazole i.v. (2-13) | 14 |
| Infection without focus | Piperacillin/Tazobactam i.v. (7-13) | 239 |
| UTI*, S. aureus* in urine | Dicloxacillin i.v. (19-25) | 43 |
| Infection without focus | Piperacillin/Tazobactam i.v. (7-13) | 25 |
| SBP, *Peptoniphilus harei* in ascites | Piperacillin/Tazobactam i.v. (2-8)* | 87 |
| Infection without focus | Piperacillin/Tazobactam i.v. (6-15) | 32 |
| Infection without focus | Piperacillin/Tazobactam i.v. (2-8)* | 8 |
| *C. difficile* infection | Vancomycin p.o. (3-12)* | Alive following 365 days |
| Infection without focus | Meropenem i.v. (10-15) and Metronidazole i.v. (10-15). | 15 |

*C. difficile, Clostridioides difficile; E. faecium, Enterococcus faecium*; i.v., intra venous; p.o., per oral; SBP, Spontaneous bacterial peritonitis; *S. Aureus, Staphylococcus aureus*; UTI, Urinary tract infection;

* Started prior to fecal sampling

**Supplementary table 4. Treatment of severe Alcohol-associated hepatitis**

| Cytolysin-positive | GAHS-score | Treatment with corticosteroids | Days of treatment, (days of treatment following hospitalization) | Lille-Score | Survival, days |
| --- | --- | --- | --- | --- | --- |
| No | 9 | Yes | 28 (10-38) | 0.16 | 338 |
| No | 10 | No^1^ |  |  | Alive following 365 days |
| No | 9 | Yes | 84^2^ (7-90) | 0.17 | Alive following 365 days |
| No | 10 | Yes | 28 (1-28)* | 0.22 | Alive following 365 days |
| No | 9 | Yes | 7 (4-10) | 0.62 | 39 |
| Yes | 11 | No^3^ |  |  | 239 |
| No | 10 | Yes | 7 (6-12) | 0.84 | 14 |
| Yes | 9 | No^4^ |  |  | Alive following 365 days |
| No | 10 | Yes | 7 (3-9)* | 0.98 | 25 |
| No | 9 | No^5^ |  |  | 87 |
| No | 10 | Yes | 1^6^ (3)* |  | 8 |
| No | 9 | Yes | 7 (2-8)* | # | Alive following 365 days |
| No | 10 | No^7^ |  |  | Alive following 365 days |
| No | 11 | Yes | 7 (3-9)* | 0.92 | 15 |

^1^ Corticosteroids decided not to be used due to severe psychiatric comorbidity

^2^ Tapering of prednisolone with 5 mg per week following 28 days of 40 mg daily

^3^ Patients refuses to receive corticosteroids

^4^ No explanation for not initiating treatment in medical record

^5^ No treatment due to ongoing intraabdominal infection (SBP, Peptoniphilus harei in ascites)

^6^ Stopped due to worsening of symptoms in the form of variceal bleeding and impaired consciousness

^7^ Shared decision-making with patient where it is decided to await spontaneous improvement following one day. Since this is achieved (GAHS decreasing to 8) treatment is not started.

* Started prior to fecal sampling

# Not measured properly due to patient leaving department against doctors’ orders, why blood samples are not drawn.
